# Structural characterization and antibacterial activity of silver nanoparticles synthesized using a low-molecular-weight Royal Jelly extract

**DOI:** 10.1038/s41598-022-17929-y

**Published:** 2022-08-18

**Authors:** Susanna Gevorgyan, Robin Schubert, Sven Falke, Kristina Lorenzen, Karen Trchounian, Christian Betzel

**Affiliations:** 1 Department of Biochemistry, Microbiology and Biotechnology, Yerevan State University, Alex Manoogian 1, 0025 Yerevan, Armenia; 2 The Hamburg Centre for Ultrafast Imaging (CUI), University of Hamburg, Luruper Chaussee 149, 22761 Hamburg, Germany; 3 Institute of Biochemistry and Molecular Biology, Laboratory for Structural Biology of Infection and Inflammation, University of Hamburg, c/o DESY, Notkestrasse 85, Build. 22A, 22607 Hamburg, Germany; 4 European X-Ray Free Electron Laser GmbH, Holzkoppel 4, 22869 Schenefeld, Germany; 5 Center for Free-Electron Laser Science (CFEL), Deutsches Elektronen Synchrotron (DESY), Notkestrasse 85, 22607 Hamburg, Germany

**Keywords:** Microbiology, Nanoparticles, Nanoparticles

## Abstract

In recent years silver nanoparticles (Ag NPs) gained increased and widespread applications in various fields of industry, technology, and medicine. This study describes the green synthesis of silver nanoparticles (Ag NPs) applying a low-molecular-weight fraction (LMF) of Royal Jelly, the nanoparticle characterization, and particularly their antibacterial activity. The optical properties of NPs, characterized by UV–Vis absorption spectroscopy, showed a peak at ~ 430 nm. The hydrodynamic radius and concentration were determined by complementary dynamic light scattering (DLS) and nanoparticle tracking analysis (NTA). The particle morphology was investigated using transmission electron microscopy (TEM), and the crystallinity of the silver was confirmed by X-ray diffraction (XRD). The antibacterial activities were evaluated utilizing Gram-negative and Gram-positive bacteria and colony counting assays. The growth inhibition curve method was applied to obtain information about the corresponding minimum inhibitory concentrations (MIC) and the minimum bactericidal concentrations (MBC) required. Obtained results showed that (i) the sizes of Ag NPs are increasing within the increase of silver ion precursor concentration, (ii) DLS, in agreement with NTA, showed that most particles have dimensions in the range of 50–100 nm; (iii) *E. coli* was more susceptible to all Ag NP samples compared to *B. subtilis*.

## Introduction

The use of honeybee products in food, medicine, and cosmetics dates to 4500 bc. These include honey, propolis, honey bread, bee venom, bee pollen, and royal jelly (RJ)^1,2^. Nowadays, bee products have historical and modern applications as remedies for human and animal diseases^1^. The increasing interest of consumers in natural products and their bioactive components with health-promoting effects derived from honeybees led to the development of new approaches for large-scale production of those components, including the use of genetically modified bee species^3^. RJ has generated considerable interest among various bee products due to its unique properties. This highly nutritional honeybee product is known for multiple benefits for human health^2,4^. Since ancient times, RJ has been used in traditional medicine, especially in Asiatic apitherapy and in ancient Egypt^5^.

RJ is a yellowish and gelatinous fluid produced by worker bees' hypopharyngeal and mandibular glands (*Apis mellifera*)^6,7^. It serves as nutrition for the larvae of worker bees and drones for only three days in their maturation process, whereas the queen honeybee is fed RJ throughout its entire life cycle^2,6^. RJ’s chemical composition and natural variation of bioactive compounds depend upon the composition of plant species present in the different ecosystems and vary corresponding to honeybee species, physiological state of the colony, environmental conditions, and production period^8,9^. Generally, RJ has been found to be mainly composed of water (50–70%), proteins (9–18%), carbohydrates (7–18%), lipids (3–8%), minerals (Fe, Na, Ca, K, Zn, Mg, Mn, and Cu), amino acids (eight essential amino acids Val, Leu, Ile, Thr, Met, Phe, Lys, and Trp), vitamins (A, B complex, C, and E), polyphenols, free fatty acids and hormones^10,11^. The pH of fresh RJ usually ranges between 3.6 and 4.2^4,12^. The variety of pharmacological properties of RJ are attributed to its unique and rich composition of proteins, carbohydrates, vitamins, lipids, minerals, flavonoids, polyphenols, and several other biologically active substances^4,13,14^. Various studies have reported antioxidant^11^, anti-inflammatory^5,15^, anti-aging^10^, neuroprotective^4^, antimicrobial^16^, antifungal^2^, anti-allergic^17^, antitumoral^13,18^, immunomodulatory^10,19^, antidiabetic^20,21^, anti-hypertension^5^ and, wound-healing^22^ properties. As a result, RJ has been used as a supplement in a number of food, cosmetic, and pharmaceutical industries^6,12^. RJ was also applied in the green synthesis of metal nanoparticles (NPs) in several studies^23,24^.

In recent years, the increase of awareness towards applying green chemistry and other biological mechanisms for the environmentally friendly synthesis of metal or metal oxide NPs contributed to research advancement in this field. Green synthesis (GS) is potentially advantageous over approaches in classical synthesis technology as it employs non-toxic and natural products to prepare metal NPs^23,25^. The concept of GS of NPs involves avoiding the use of harmful and toxic reducing agents, such as hydrazine hydrate, sodium borohydride, and various other chemicals^26,27^. GS of metal NPs is based on a redox reaction when metal ions are reduced to form a stable NP. Living organisms, such as bacteria, fungi, algae, and plants, and their extracts have been applied to synthesize metal NPs with highly diverse morphology^28^. In this way, *Rubia cordifolia* leaf-extract-mediated bio-fabricated silver NPs demonstrated significant cytotoxic activity against skin cancer cell lines^29^, whereas ZnO NPs, synthesized using *Muntigia carabula* leaf extract, showed efficiency in photocatalytic degradation of a specific molecule used as herbicide^30^. Cellulase, isolated from *Glutamicibacter arilaitensis*, was used in the biosynthesis of Au NPs with a promising application in saccharification process^31^. Another study showed that protein-capped CdS NPs inhibited Tau protein fibrilization^32^. The NPs are endowed with unique physicochemical properties and biological activity due to their small size and large surface-to-volume ratio^3,33,34^. These characteristics make them valuable for applications in various fields of industry, medicine, and technology^3^. Therefore, there is need for the development of sustainable approaches.

The green chemistry-based approach gained substantially more popularity due to its simplicity, low cost, environmental friendliness, and the possibility of scaling up^35^. In our recent work^23^, raw RJ's aqueous solution was applied in the GS of silver nanoparticles (Ag NPs). However, the stability of the obtained NPs was low, in agreement with the presence of large, clustered particles in a suspension, which led to the formation of visible precipitate over time and changes in biological activity. Based on the previous investigation, in order to improve the quality and stability of Ag NPs, the current study aimed (i) to investigate the potential of the low-molecular-weight fraction extract of RJ in the GS of Ag NPs, (ii) to examine the influence of various concentrations of silver ion precursor on properties of Ag NPs, (iii) to identify their physicochemical characteristics and (iv) to assess their antibacterial activity on Gram-negative and Gram-positive bacterial strains.

## Materials and methods

### Materials

Raw royal jelly (RRJ) was supplied by the local Armenian beekeeping factory ‘’Royal Jelly’’ LTD (Province Kotayk, Armenia) and stored at – 20 °C. Silver nitrate was purchased from Carl Roth (Germany). The Luria–Bertani (LB) broth and other media components were purchased from Sigma-Aldrich and Carl Roth (Germany). All solutions were prepared in deionized water.

### Preparation of Ag NPs

**Figure 1:**
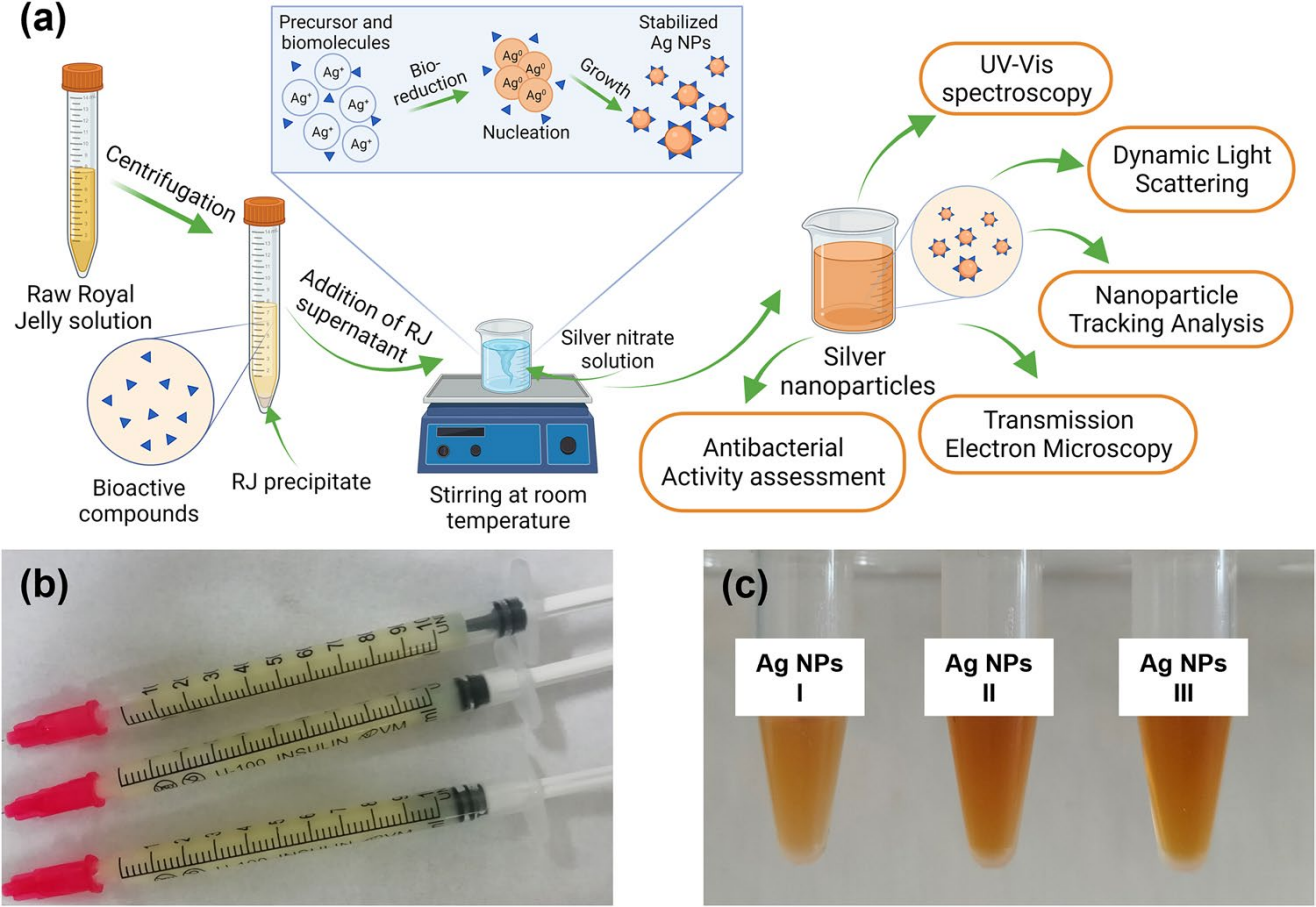
Scheme of the green synthesis of Ag NPs using Royal Jelly (**a**), Raw Royal Jelly (**b**) and GS Ag NPs (**c**).

Ag NPs were prepared by reducing Ag^+^ ions using RJ extract as a source of reducing agents. The aqueous solution of RRJ in 20 mg mL^−1^ concentration was centrifuged at 4500*g* for 40 min (Eppendorf, 5804R, Germany). Transparent supernatant was used in the subsequent synthesis process (Fig. 1). Ag NPs were synthesized by mixing RJ supernatant at a 1:1 volume ratio with 1 mM, 2.5 mM, and 5 mM solutions of AgNO_3_, respectively, and stirring the mixture for 20 min before keeping under lamplight (~ 230 V, 50 Hz, 11 W) for 6 h at room temperature. The NPs samples synthesized using 1 mM, 2.5 mM, and 5 mM silver nitrate solutions, were labelled in order as follows: Ag NPs-I, Ag NPs-II, Ag NPs-III. Further characterization and antibacterial activity assessment were conducted on all Ag NP samples as synthesized.

### Ag NPs characterization

The formation of Ag NPs was confirmed by UV–Vis absorption spectra with a wavelength range of 280–780 nm at a resolution of 1 nm using a Nanodrop 2000C Spectrophotometer (Thermo Scientific, USA). Each sample of GS Ag NPs was diluted in deionized water, and 50 μL volume of each suspension was loaded into a cuvette (UVette; Eppendorf, Germany) and applied for UV–Vis absorption spectrum measurements.

The dynamic light scattering method was employed for the determination of hydrodynamic radii. Each sample of NPs diluted in deionized water was analysed using a SpectroSize 300 instrument (XtalConcepts, Germany) at 660 nm wavelength. 15 μL of each sample was loaded into a closed quartz high precision cell (Hellma Analytics, Germany; light path: 1.5 × 1.5 mm). Twenty measurements each for 20 s duration were recorded, and the autocorrelation functions were analysed by the CONTIN algorithm^36^.

The hydrodynamic diameter and concentration of GS Ag NP suspensions were determined by a Nanosight LM10 nanoparticle tracking instrument (Malvern Panalytical, UK) injecting a diluted suspension volume of 300 μL of each sample into the flow cell. All dilutions were prepared in deionized water. Five particle tracking periods with 20 s duration each were recorded applying a 405 nm wavelength laser. The obtained data were averaged and normalised using the accompanying software.

To determine the colloidal stability of GS Ag NPs, the Mobius device (Wyatt Technology, USA) was used to measure the zeta potential of five-fold diluted aqueous suspensions of the GS Ag NPs. The flow cell was loaded with ~ 180 µL of each sample. A 532 nm laser, 2.5 V voltage amplitude, and 10 Hz electric field frequency were used to record three simultaneous DLS and Phase Analysis Light Scattering (PALS) measurements with a duration of 5 s each. A total of 31 independent diodes collected scattered light for PALS analysis. The Smoluchowski equation function of the DYNAMICS software (Wyatt Technology, USA) was used to calculate zeta potential values. The arithmetic means of the obtained data were calculated based on three measurements.

In-depth analysis of morphology, size distribution, and aggregation of GS Ag NPs was performed by using transmission electron microscopy (TEM, JEM-2100-Plus, JEOL, Germany). For TEM measurements, a single drop of each sample was placed on freshly negatively charged quantifoil copper grids (R1.2/1.3, 400 mesh, Science Services, Germany), incubated, blotted, and dried. Transmission electron microscopy images were recorded at an accelerating voltage of 200 kV. Additionally, selected area electron diffraction patterns (SAED) were collected. TEM experiments were conducted in the XBI Biolab of the European XFEL^37^.

X-ray diffraction (XRD) experiments were performed using a MAR345 image plate detector (detector diameter: 345 mm; pixel size: 0.15 mm; MARXperts GmbH, Germany) coupled with a micro-X-ray tube (IµS, INCOATEC GmbH, Germany) providing Cu-Kα radiation (λ = 1.54179 Å) at 50 kV and 1 mA current. Data were collected applying a dried sample on a silicon chip (Suna-Presicion GmbH, Germany) with 600 s exposure time and 10° rotation per image, and a sample to detector distance of 76 mm. Sample crystallinity was determined by comparing observed patterns with standard powder patterns of the Joint Committee on Powder Diffraction Standards (JCPDS).

### Bacterial strains, culture conditions, and growth determination

The present study was performed using Gram-negative and Gram-positive bacteria. Two wild-type strains were applied: *Escherichia coli* BL-21 and *Bacillus subtilis* sp. 168. Bacterial strains were kindly provided by Prof. Daniel Wilson (Institute of Biochemistry and Molecular Biology, University of Hamburg, Germany).

*E․ coli* and *B. subtilis* were cultivated in an LB broth medium (pH = 7.5) at 37 °C. The pH of the medium was measured with a pH-selective electrode (Mettler Toledo, Switzerland) and adjusted by 0.1 M NaOH or HCl. Calculation of the specific growth rates after inoculation of the medium with bacteria was conducted using the equation below:

1 $$\mu =\frac{{\mathrm{ln}(OD)}_{2}- {\mathrm{ln}(OD)}_{1}}{{t}_{2}- {t}_{1}},$$

where *µ* is the specific growth rate (h^−1^), *OD*_*1*_ and *OD*_*2*_ are two optical density (OD) values chosen on the growth timeline at a wavelength of 600 nm, corresponding to *t*_1_ and *t*_2_ time points^38^.

### In vitro susceptibility test

Preliminary antibacterial activity screening of GS Ag NPs against selected bacteria was tested by the Kirby-Bauer Disk Diffusion (DD) susceptibility method with some modifications^39^. Overnight grown bacteria strains were spread on LB-agar using a sterile cotton swab. A sterile blank disk was used in the test. The disks were prepared as follows: filter paper disks with 6 mm diameter were sterilized by autoclaving and then loaded with 20 μL RJ LMF extract and Ag NPs samples. After spreading the bacteria on the LB-agar surface, the disks were placed on the agar plates and incubated at 37 °C for 24 h. The inhibition zone was documented and measured after 24 h of incubation. The experiments were done in two replicates for each strain.

### Evaluation of minimum bactericidal concentration (MBC) and minimum inhibitory concentration (MIC)

The MIC and MBC of GS Ag NPs were examined as described by Loo et al.^25^ with some modifications. The standard broth microdilution method was applied to perform MIC determination in 96-well microtiter plates. Prior to the MIC test, Ag NPs-II and Ag NPs-III samples were diluted to the same concentration as Ag NPs-I (54 µg mL^−1^). Each solution of GS Ag NPs samples was mixed with LB-medium at a 1:1 volume ratio and then two-fold diluted. Negative (i.e. only medium) and positive (i.e. medium and bacterial inoculums) controls were applied within the experiments. The overnight grown bacterial suspension was transferred into fresh LB-medium and diluted to an OD of ~ 0.1 measured at 600 nm wavelength (TECAN Reader Infinite 200 M Plex, Switzerland). The 96-well microtiter plates were incubated at 37 °C, and the OD changes of a bacterial suspension were recorded at 600 nm wavelength for 24 h. The bacterial specific growth rate was determined as described above (Eq. 1). MIC values were calculated by determining the lowest concentration of Ag NPs to inhibit the growth of bacteria.

The MBC test was performed on LB-agar plates to determine the lowest concentration that is bactericidal. After 24 h of growth, suspension from each well and when required their distinct dilutions (10^1^–10^8^), were applied for determining the colony-forming ability by the drop plate (DP) method^40,41^. 10 μL of each sample was transferred onto LB-agar plates and incubated at 37 °C. After 24 h of incubation, viable bacterial colonies were counted to determine the number of colony-forming units (CFUs). NP-free plates as controls were incubated under the same conditions. MBC values are determined by the lowest concentration in which no visible growth appears on LB-agar plates.

### Data processing and statistical analysis

The average data and calculated standard deviations (SDs) are presented based on 3 independent experiments; if not shown, they do not exceed 3%. Multiple groups were compared by one-way analysis of variance (ANOVA); unless otherwise specified, p < 0.05 or less. ImageJ software was used to process TEM micrographs. Figure 1a and 7 were prepared using Biorender.com.

### Ethics declarations

This article does not contain any studies with human and animal participants.

## Results and discussion

### UV–Vis spectra analysis

**Figure 2:**
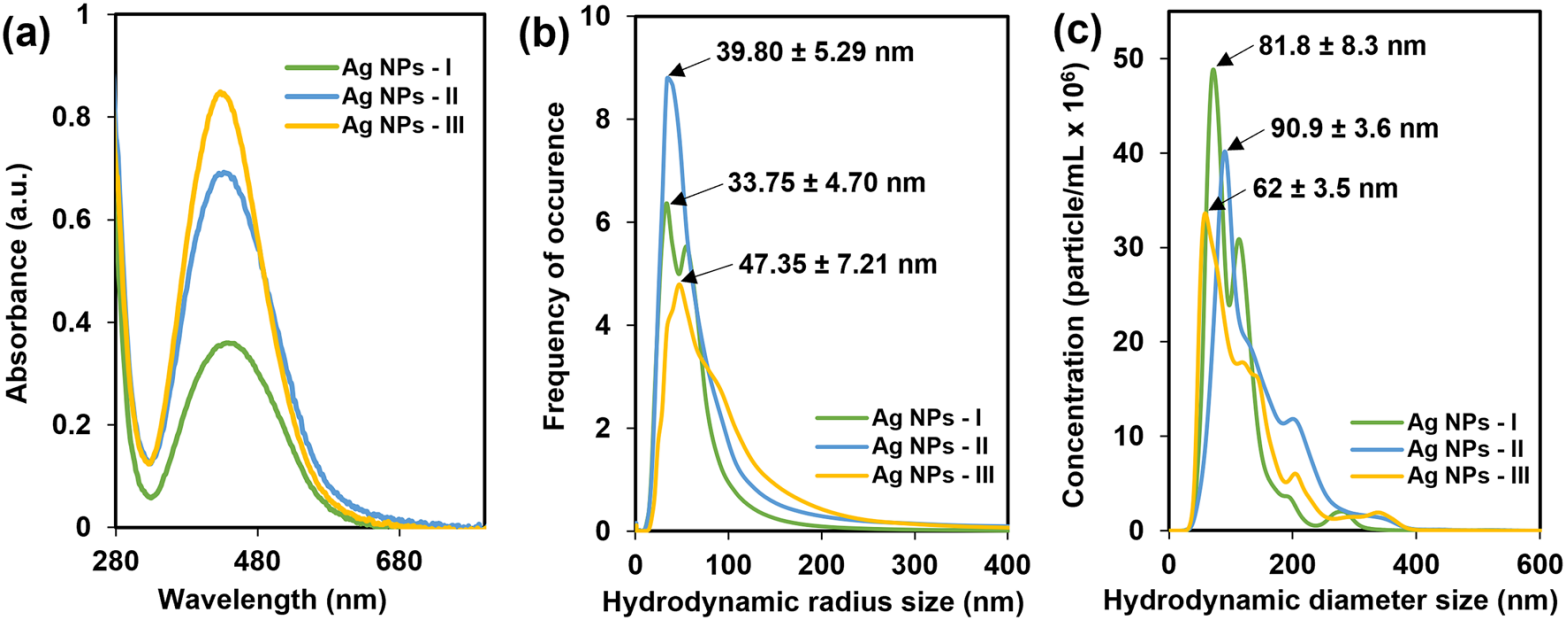
UV–Vis spectra (**a**), hydrodynamic radii measured by DLS (**b**), and correlation of hydrodynamic diameter and concentration determined by NTA (**c**).

As the first level of GS NP characterization, UV–Vis spectroscopy is a relatively fast, simple, and sensitive method^42^. Color changes of the reaction mixture indicated the reduction of Ag^+^ ions and the formation of Ag NPs^43^. A brownish color of the solution attributed to Ag NPs is conditioned by the surface plasmon resonance (SPR) that results from an oscillation of electrons when they are in resonance with light waves^42^. Ag NPs are known to exhibit a UV–Vis absorption maximum in the range of 400–500 nm^44^. The absorption spectra of GS Ag NPs are shown in Fig. 2a. Obtained results revealed a strong SPR band maximum at ~ 430 nm, which is characteristic for Ag NPs^25,45^. During the synthesis, the transparent solution changed its color gradually to brownish, indicating the reduction of Ag^+^ ions and formation of Ag NPs, in that the higher the concentration of AgNO_3_, the darker the color of the final solution, and the absorbance was comparatively higher. The SPR peak is sensitive to particle size and shape as well as dielectric medium and surroundings^42^. The absorbance peak shifts towards longer wavelengths indicate the increase in particle size^46^.

### Dynamic light scattering

Physicochemical characterization of GS NPs is an essential step before studying biological activities. Among various techniques for the characterization of NPs, the most used is DLS^42^. DLS is a non-destructive light scattering analytical method widely applied in different fields of life and material sciences^47^. The method depends on the interaction of light with particles and is based on the measurements of light intensity fluctuations over time due to particle Brownian motion^42,47^. This allows for determining the diffusion coefficient (D), which is related to the hydrodynamic radius (R_h_) of the particle through the Stokes–Einstein equation,

2 $$D=\frac{{k}_{b}T}{6\pi \eta {R}_{h}},$$

where ${k}_{b}$ is the Boltzmann constant (1.380 × 10^–23^ kg m^2^ s^−2^ K^−1^), *T* is the absolute temperature, and *η* is the viscosity of the medium^47^.

The particle size distribution data from DLS measurements are summarized in Fig. 2b. DLS results showed polydispersity in each sample. Obtained results revealed that the hydrodynamic radius of NPs is mainly in the range of 30–100 nm. In this range, the radius increased with the increase of silver nitrate concentration in the applied solution. The samples' polydispersity index (PDI) was ~ 32% up to ~ 34%, confirming a relatively broad particle size distribution of the sample suspensions.

### Nanoparticle tracking analysis

NTA is a frequently applied technique for particle size and concentration determination in liquid samples. Laser light scattering microscopy in combination with a charge-coupled device (CCD) camera enables visualization and recording of particles in a solution^48^. Individual particles moving under Brownian motion are identified and tracked to determine their speed of movement. The software calculates the hydrodynamic diameter of particles based on a modified Stokes–Einstein equation and also provides an approximate concentration of particles per volume^49^ (Fig. 2c). In agreement with DLS measurement results, NTA showed that most particles are in size range of 50–100 nm. The number of particles per sample increased between Ag NPs-I (dilution: 1:25) and Ag NPs-II (dilution: 1:50), while it was relatively constant comparing Ag NPs-II and Ag NPs-III (dilution: 1:50). Averaged values with standard error of the particle concentration are presented in Table 1.

**Table 1:** Averaged concentration of GS Ag NPs determined by NTA.

Sample	Concentration (particle/mL × 10^9^)	SE (× 10^7^)
Ag NPs-I	3.28	± 6.34
Ag NPs-II	3.60	± 1.18
Ag NPs-III	3.14	± 2.79

### Zeta potential evaluation

Zeta potential determination was performed to assess the stability of particles in a suspension based on their surface charge^50^. A large positive and large negative zeta potential value indicates high stability of the particles conditioned by substantial repulsive forces that also prevent aggregation^51^. NPs with zeta potential values of less than + 25 mV and greater than − 25 mV tend to form aggregates mediated by the interparticle interactions^52^. Zeta potential measurements showed that all GS Ag NPs are positively charged with values of 24.1 ± 0.7 mV, 27.7 ± 0.7 mV, and 28.1 ± 2.6 mV for Ag NPs-I, Ag NPs-II and Ag NPs-III, respectively. Accordingly, the stability of NPs was increasing with the increase of the applied precursor concentration.

### Transmission electron microscopy

**Figure 3:**
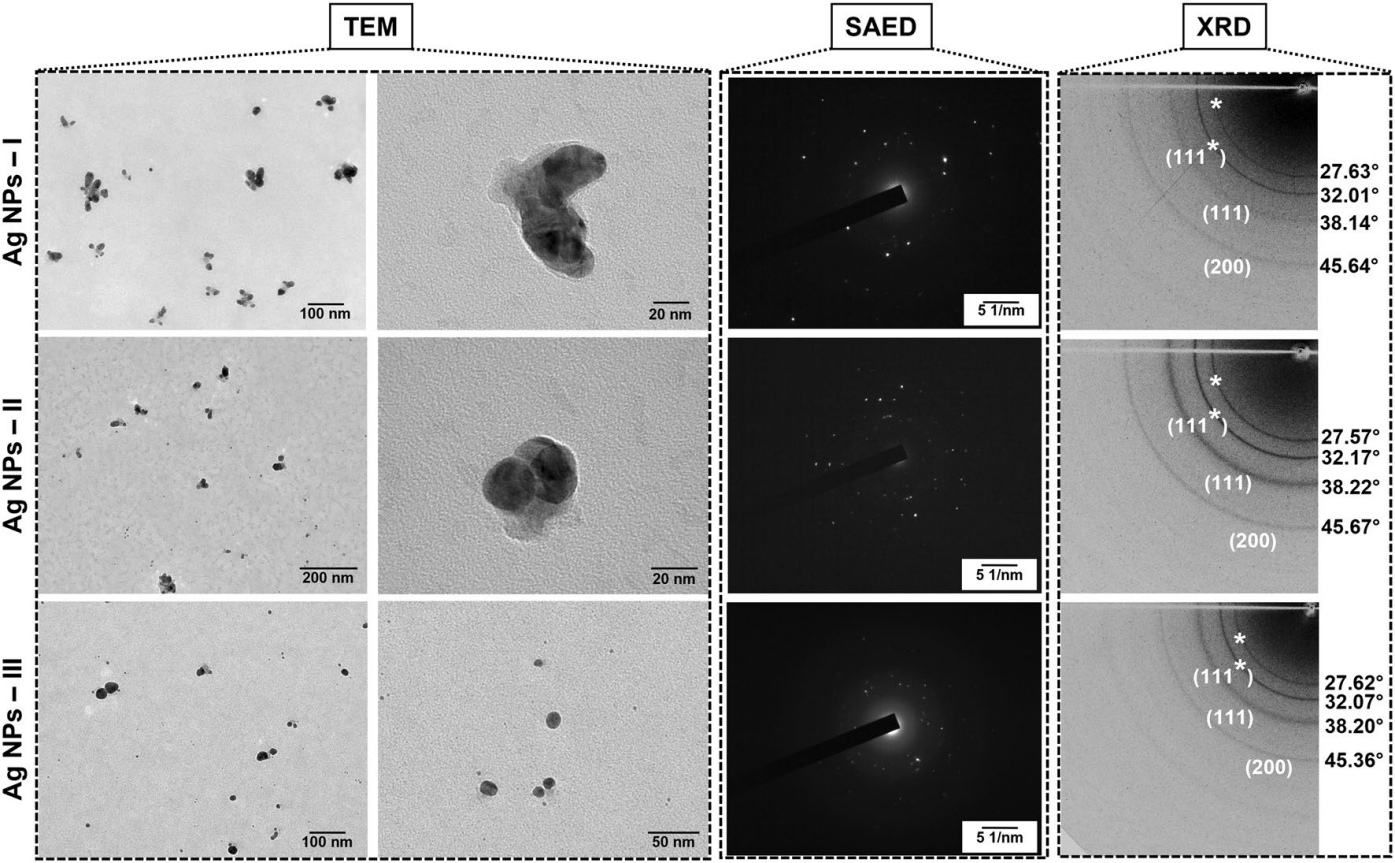
Imaging of Ag NPs. TEM micrographs, SAED and XRD patterns of Ag NPs synthesized under different concentrations of silver nitrate: 1 mM (Ag NPs-I); 2.5 mM (Ag NPs-II); and 5 mM (Ag NPs-III).

TEM is a frequently used technique for the characterization of morphology and size of nanomaterials^42^. TEM micrographs shown in Fig. 3, revealed that all samples of GS Ag NPs contain single as well as clustered particles. The Ag NPs-I sample contained more branched or clustered particles compared to the other sample suspensions. The size and growth of clusters are conditioned and controlled by interfacial chemical reactions and particle transport mechanism^53^. Many studies evidence that smaller particles are more susceptible to aggregation^54^. As the particle size decreases, the surface energy increases, causing a change in surface reactivity and system destabilization^55^. The aggregation will lower the systems' free energy, thus stabilizing it^53^. Another crucial factor for the growth of particles is mass transfer. It is controlled by the rate of stirring of the reaction mixture^56^. Various studies showed that stirring rate could reduce NP synthesis duration, but on the other hand, high stirring velocities can increase the temperature and promote thermal oxidation of particles^57^.

In addition to TEM results, SAED (Fig. 3) confirmed the crystalline nature of GS Ag NPs.

### XRD analysis

The examination of all GS Ag NP samples by XRD confirmed their crystallinity. The 2θ angles and corresponding hkl planes shown in Fig. 3 are indexed to the face centred cubic (fcc) structure of metallic silver (JCPDS file no. 84-0713 and 04-0783)^58,59^. Additionally, for all three samples of NPs two strong diffraction rings were observed corresponding to 2θ angles of ~ 28° and ~ 32°. The angle of ~ 32° can be linked to silver oxide (plane (111*), Fig. 3)^60^, while an angle of ~ 28° (marked with stars, Fig. 3) may be attributed to the organic composition of GS Ag NPs. It might be assumed that the higher intensities of the unassigned rings corresponding to an angle of ~ 28° indicate a relatively high content of ordered organic molecules compared to silver. A number of previous studies on green synthesis of Ag NPs reported comparable XRD results^61,62^.

### Antibacterial activity assessment

**Figure 4:**
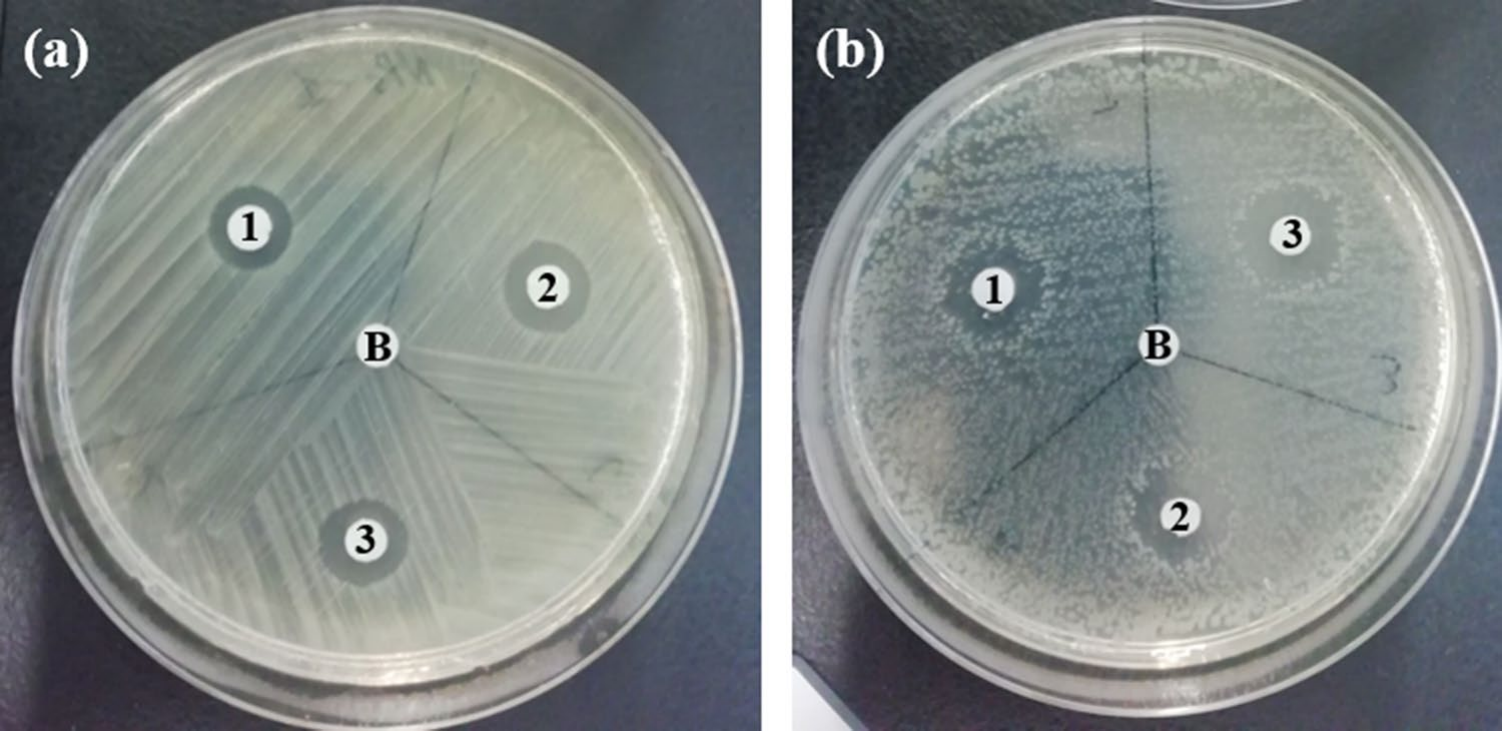
Disk diffusion assays showing zones of inhibition of Ag NPs-I (1); Ag NPs-II (2); and Ag NPs-III (3) on *E. coli* BL-21 (**a**) and *B. subtilis* sp. 168 (**b**).

The GS Ag NPs revealed varying levels of antibacterial activity on both Gram-negative and Gram-positive bacteria depending on their dimensions and aggregation state. Significant antibacterial activity has been observed in the case of Ag NPs-III. The results of the DD experiments are summarized in Fig. 4. Ag NPs possessed antibacterial activity on both *E. coli* and *B. subtilis* bacteria as evidenced by a clear zone surrounding the disk.

**Figure 5:**
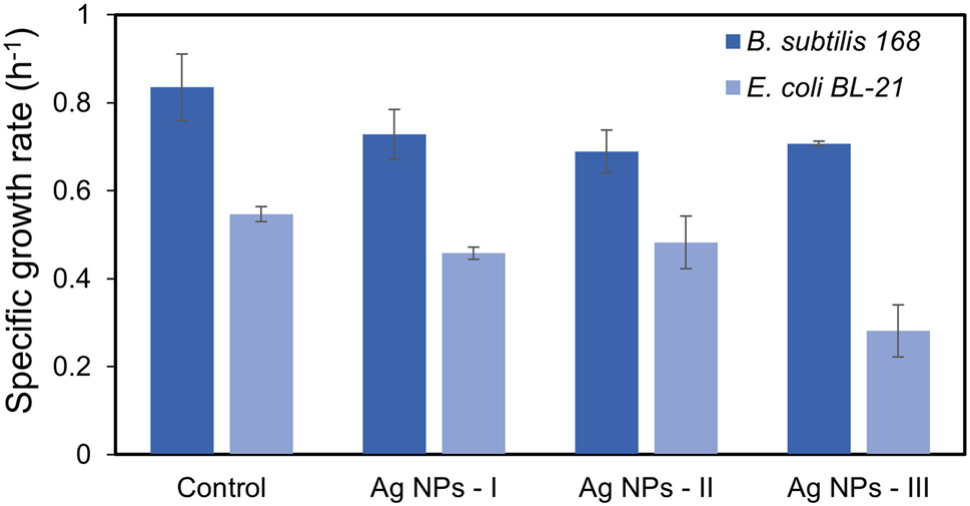
Specific growth rates of *E. coli* at 6.75 µg mL^−1^ and *B. subtilis* at 27 µg mL^−1^ concentration of all NPs.

**Figure 6:**
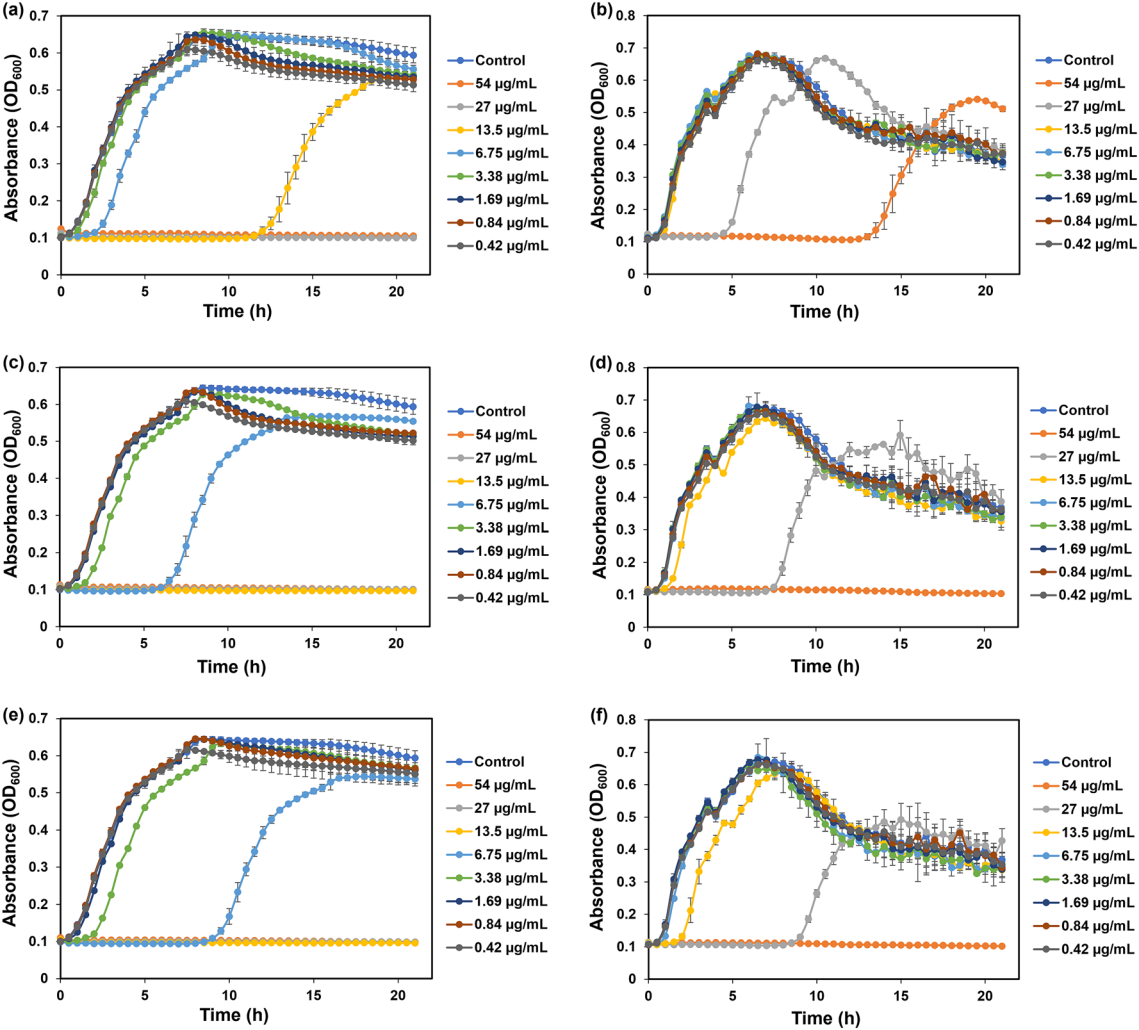
Growth curves of *E. coli* (**a**,**c**,**e**) and *B. subtilis* (**b**,**d**,**f**) with addition of Ag NPs-I (**a**,**b**), Ag NPs-II (**c**,**d**), Ag NPs-III (**e**,**f**).

The preliminary experiments to evaluate the antibacterial activity of GS Ag NPs were DD experiments. A further investigation in estimating the antibacterial activity was performed by determining the MIC and MBC. The MIC of Ag NPs was defined as the lowest concentration at which significant inhibition of bacterial growth was achieved. *E. coli* revealed an MIC value of 13.5 µg mL^−1^ for Ag NPs-I and 6.75 µg mL^−1^ for both Ag NPs-II and Ag NPs-III, in that more potent inhibition has been observed in the case of Ag NPs-III. Ag NPs-II inhibited the growth of bacteria for ~ 6 h, after which the bacteria started to recover, while in the case of Ag NPs-III the growth was inhibited for ~ 9 h (Fig. 6). This difference can be conditioned by the presence of clustered particles in Ag NPs-II, whereas Ag NPs-III contained more single particles which demonstrate higher reactivity and antibacterial activity. The MIC for *B. subtilis* was 27 µg mL^−1^ for all three suspensions of Ag NPs, in that inhibition duration was increased from Ag NPs-I to Ag NPs-III. The specific growth rates expressed in h^−1^ and calculated for the exponential growth interval for both bacteria are summarized in Fig. 5.

The lowest concentration of NPs that was bactericidal, i.e. that showed no growth on agar plates, was selected as MBC. In this way, for *E. coli* in case of Ag NPs-I the MBC was 27 µg mL^−1^ and 13.5 µg mL^−1^ for both Ag NPs-II and Ag NPs-III. In case of *B. subtilis* Ag NPs-II and Ag NPs-III showed bactericidal activity at 54 µg mL^−1^ concentration, while Ag NPs-I exhibited only an inhibitory effect, since the growth was recovered (Fig. 6).

Results showed that *E. coli* is more susceptible to all Ag NP samples prepared than *B. subtilis*. Ag NPs-III demonstrated higher antibacterial activity compared to other sample suspensions of NPs. This can be conditioned by the presence of small-sized particle fractions, which can easily penetrate through the cell wall and cause disorders in cell functions such as permeability, respiration, and apoptosis^63^. Antimicrobial properties of silver and its organic and inorganic compounds against a wide-range of microorganisms, have been well-known for centuries dating back to ancient times^64,65^. The increasing rate of multiple antibiotic resistance among bacterial strains to traditional antibiotics, including penicillin, biomycin, and others, prompted the development of Ag NPs as antibacterial agents in recent years^63,66^. Various studies showed that the size, shape, aggregation, or agglomeration of Ag NPs, which are mainly dependent on the synthesis conditions of NPs, i.e. temperature, silver ion precursor concentration, pH, etc., play a crucial role in their toxicity and biological activity^67^.

**Figure 7:**
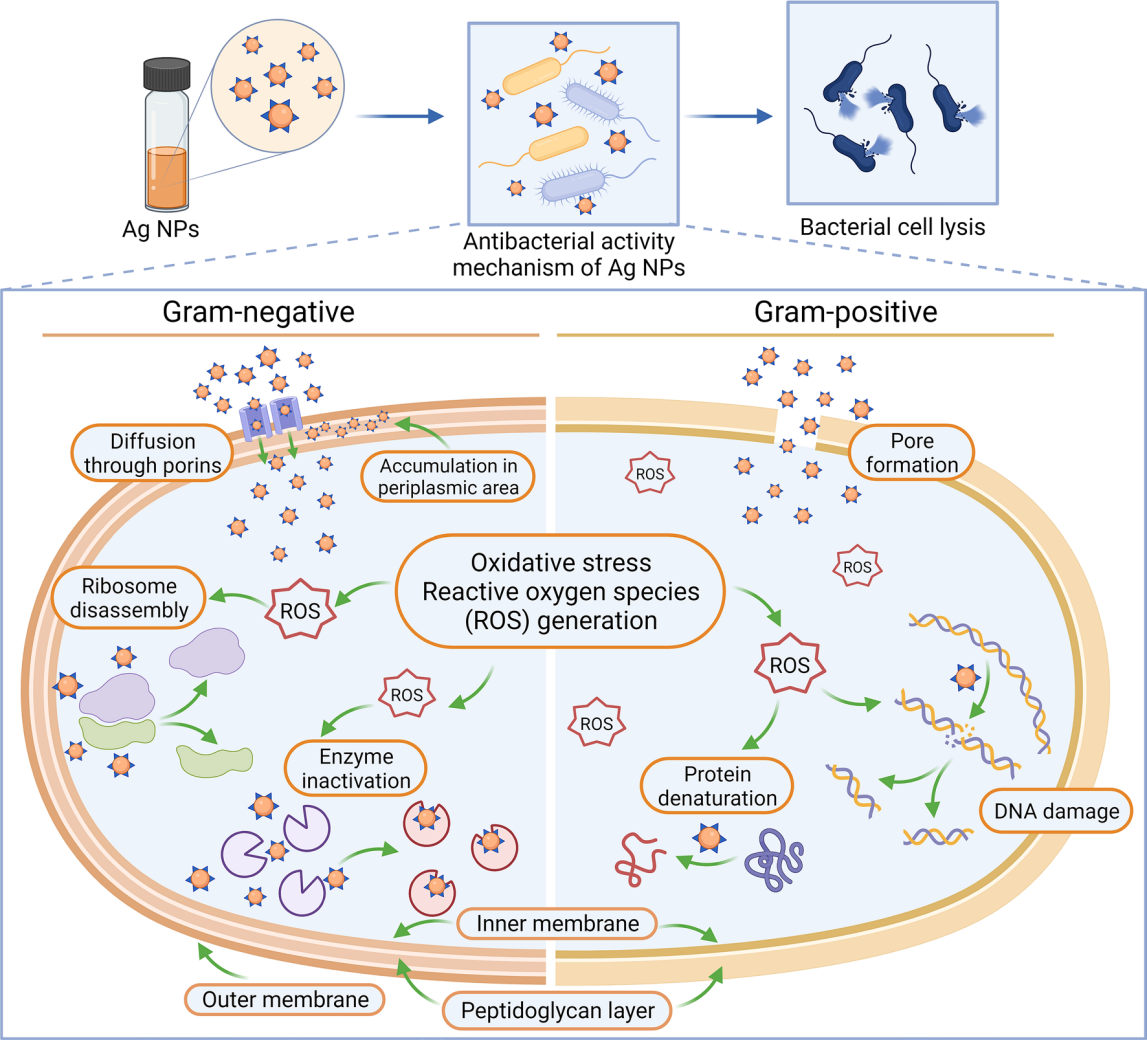
Schematic representation of possible antibacterial activity mechanism of Ag NPs on Gram-negative and Gram-positive bacteria prepared based on available data in scientific literature^63,68,69^.

Even though many publications have been devoted to understanding the mechanism of action of the Ag NPs, the exact process remains unclear. Figure 7 illustrates several possible mechanisms of antibacterial activity of Ag NPs. However, available data in scientific literature allows separating two main approaches regarding the action mechanism of Ag NPs. The first one is interaction with bacterial cell membranes through perforating and penetrating the cells. The NPs can accumulate on the surface and inside the bacterial cell, where they can bind to cellular machinery components, thus damaging essential cell functions and destroying the cell^63^. Besides that, Ag NPs can lead to the release of reactive oxygen species, thus leading to cell death^68^. The thinner cell membrane of Gram-negative bacteria makes them more susceptible to Ag NPs compared to Gram-positive bacteria^70^. Ag NPs can easily penetrate bacterial cells through the porins in the outer membrane of Gram-negative bacteria^63^. The second possible mechanism of action of NPs can be the release of silver ions. The NPs can attach to the cell wall due to the electrostatic interaction of positively charged Ag NPs and the negatively charged surface of the cell membrane. This kind of interaction can cause structural changes in the cell membrane, change permeability, dissipate proton motive force, and thus destroy the membrane^63^. In addition, bacterial growth inhibition can occur by depositing silver ions into the vacuole and cell walls as granules^71^. Lara et al.^69^ reported the interaction of Ag NPs and DNA compounds. According to earlier reports, Ag^+^ ions released from NPs can inhibit DNA replication due to DNA double-strand breakage and due to inactivating proteins by binding to them, potentially leading to cell death^72^. Therefore, the question of the leading player in the antibacterial effect of Ag NPs—silver ions, or NPs—is still open. However, it is evident that NPs are most valuable compounds and that the approach of designing NPs is of high interest to complement and serve as an alternative to antimicrobial drug discovery and development.

## Conclusions

The presented data and results confirm that the low-molecular-weight fraction of the RJ extract can be used for the green synthesis of Ag NPs. Complementary DLS and NTA experiments of NP suspensions revealed nanoscale size of particles. Spherical and clustered NPs were observed using TEM. The crystalline nature of all NP samples was confirmed applying SAED and XRD. Furthermore, in addition to silver content, the XRD results suggested the presence of silver oxide and organic materials. The latter can be related to the composition of low-molecular-weight fraction of RJ extract. The concentration of silver ion precursors during synthesis plays a critical role in the formation, stability, and functional activity of synthesized NPs. In the case of lower concentrations of silver nitrate, particles form larger clusters to become more stable, which however leads to particle aggregation and a decrease in their antibacterial activity. GS Ag NPs demonstrated antibacterial activity against one representative species of both Gram-positive and Gram-negative bacteria. In this context, Gram-negative bacteria were more susceptible to all applied NPs. The high and growing demand for NPs requires eco-friendly approaches to reduce the environmental footprints and preferably use of biocompatible and sustainably produced substances, which is attainable based on a cost-effective RJ-mediated synthesis approach.

## Data Availability

All data generated and/or analysed during this study are included in this article.
